# Supplementary material for: A Global Screen for Assembly State Changes of the Mitotic Proteome by SEC-SWATH-MS
Source: Cell Syst. 2020 Feb 26;10(2):133–155.e6. doi: 10.1016/j.cels.2020.01.001 (PMC7042714; doi:10.1016/j.cels.2020.01.001)

Q9Y2G5 | OFUT2_HUMAN | POFUT2 C21orf80 FUT13 KIAA0958
Monomer MW [kDa]: 49.976 Monomer expected elution fraction: 46

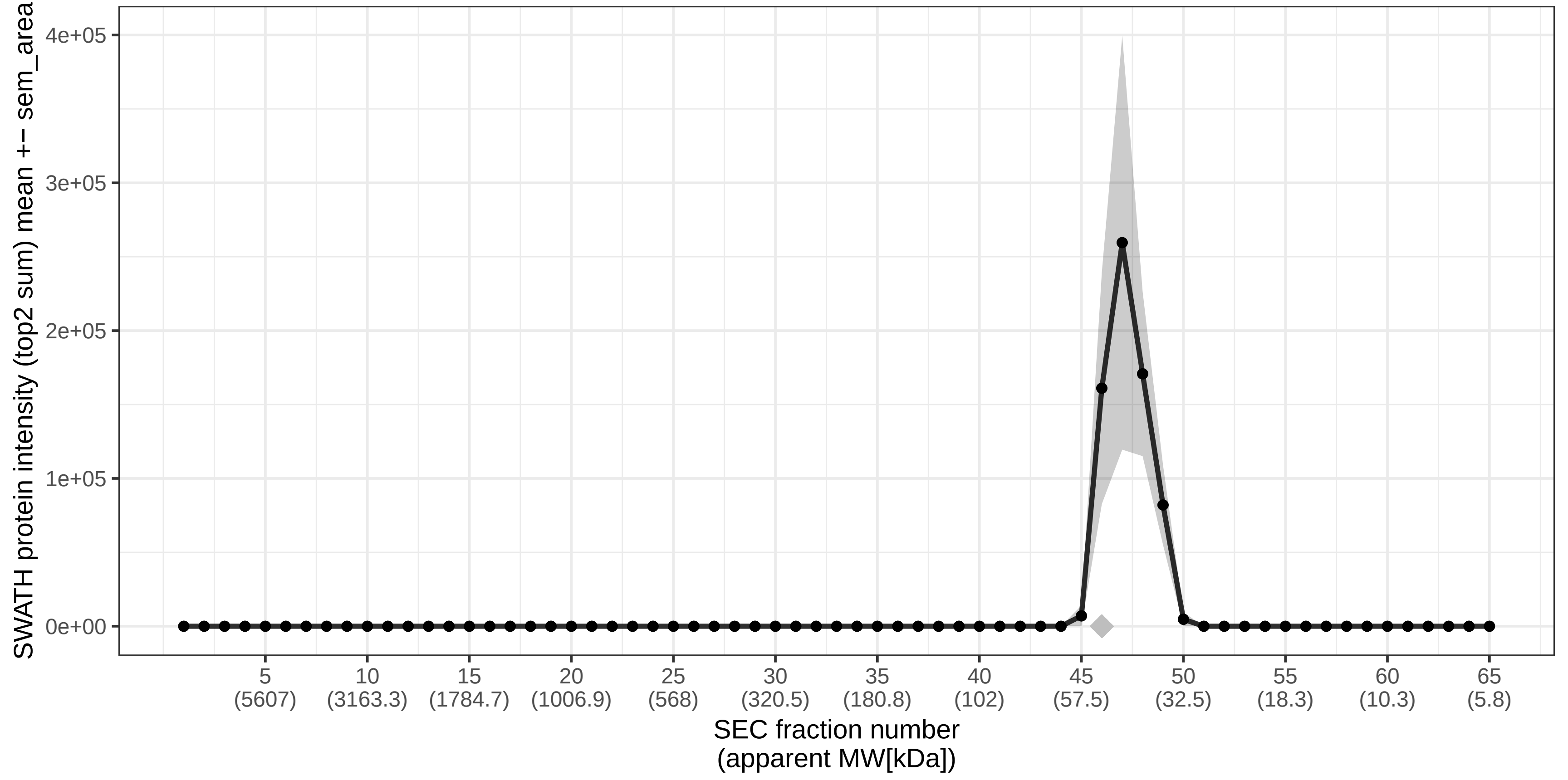

Supplement: Data S1. SEC-SWATH-MS Protein Chromatograms, Related to Figure 1 [file mmc6.zip › SECchrom_Q9Y2G5_OFUT2_HUMAN_POFUT2_C21orf80_FUT13_KIAA0958.pdf]
